# Analyzing research trends in glioblastoma metabolism: a bibliometric review

**DOI:** 10.3389/fimmu.2024.1444305

**Published:** 2024-10-18

**Authors:** Jiaxin Dai, Siyun Song, Pengyu Chen, Qixuan Huang, Hubin Duan

**Affiliations:** ^1^ First Clinical Medical College, Shanxi Medical University, Taiyuan, China; ^2^ Department of Neurosurgery, First Hospital of Shanxi Medical University, Taiyuan, China; ^3^ Department of Gastrointestinal Surgery, Harbin Medical University Cancer Hospital, Harbin, China; ^4^ Third Clinical Medical College, Harbin Medical University, Harbin, China

**Keywords:** glioblastoma, metabolize, metabolic reprogramming, tumor microenvironment, bibliometric analysis, hotspots

## Abstract

**Background:**

A bibliometric and visual analysis of articles related to glioblastoma metabolism was conducted to reveal the dynamics of scientific development and to assist researchers in gaining a global perspective when exploring hotspots and trends.

**Methods:**

The Web of Science Core Collection (WoSCC) was employed to search, screen, and download articles about glioblastoma metabolism published between 2014 and 2024. The relevant literature was analyzed using CiteSpace, VOSviewer and Microsoft Excel.

**Results:**

A total of 729 articles were included for bibliometric analysis between 2014 and 2024, and the number of articles published each year showed an overall increasing trend, except for a decrease in the number of articles published in 2018 compared to 2017. Collaboration network analysis showed that the United States, Germany and China are influential countries in this field, with a high number of articles published, citations and collaborations with other countries. The journal with the largest number of published articles is the International Journal of Molecular Sciences. Mischel PS is the most prolific author with 14 articles, and Guo DL received the most citations with 104 citations. Keyword analysis of the literature showed that the “Warburg effect” achieved the highest burst intensity, and “central nervous system”, “classification” and “fatty acids” showed stronger citation bursts in 2024, indicating that they are still popular topics so far.

**Conclusion:**

This article elucidates the research trends and focal points in the field of glioblastoma metabolism, furnishes invaluable insights into the historical and contemporary status of this field, and offers guidance for future research. Further research into glioblastoma metabolism will undoubtedly yield new insights that will inform the diagnosis and treatment of this disease.

## 1 Introduction

Glioblastoma (GBM) is a common malignant tumor in humans, with a high recurrence, mortality, aggressiveness, proliferation, and recurrence rate (1–3). Due to the critical function of the brain and the metastatic nature of GBM making surgical resection complex and challenging, coupled with patient resistance to temozolomide and the protective effect of the blood-brain barrier against tumors, even with the current standard of care, which consists of a combination of surgical resection, radiotherapy and chemotherapy with temozolomide, the outcome of patients with GBM is not satisfactory, with the median survival is only 8 months and a merely five-year survival rate of 6.9% (4–7).

More than 100 years ago, the German biochemist Otto Warburg discovered the metabolic differences between tumor cells and normal tissues and proposed the famous “Warburg effect” (8). This discovery was one of the major milestones in cancer research in the 20th century. Wahlberg observed that tumor cells tend to produce more energy through glycolysis than normal cells, even in the presence of sufficient oxygen (9). This discovery revealed the fundamental characteristics of tumor metabolism and provided important clues for subsequent cancer research and treatment.

As is the case with the majority of cancers, GBM cells undergo metabolic reprogramming for various growth- and survival-promoting functions, playing a critical role in GBM progression and recurrence (10). In GBM cells, common metabolic abnormalities include aberrations in lipid (11–14), amino acid (15–18) and nucleotide metabolism (19), as well as enhanced glycolysis (20–23). For example, the research team led by P. Chen discovered that the lactate dehydrogenase A (LDHA)-mediated extracellular signal-regulated kinase (ERK) pathway can activate the transcriptional coactivators YAP1 and STAT3 in GBM cells, thereby upregulating the expression of CCL2 and CCL7. GBM cells induce macrophages to infiltrate the tumor microenvironment by upregulating CCL2 and CCL7, and then macrophages promote tumor growth and survival by delivering LDHA to tumor cells (24). The present study proposes a novel strategy for the treatment of GBM, which targets the LDHA-mediated tumor-macrophage symbiosis. Given that these metabolic adaptations may be present in multiple genotypes, therapeutic strategies targeting aberrant metabolism may lead to new ideas for treating GBM in the clinic.

The intricate and diverse nature of the GBM immune microenvironment has historically posed a significant challenge in scientific research. Historically, the lack of effective tools for investigating TAMs in GBM has constrained our understanding of GBM heterogeneity (25, 26). However, with the ongoing advancement of artificial intelligence and machine learning technologies, coupled with the persistent efforts of scientific researchers, the study of tumor heterogeneity has made a significant advancement. Artificial intelligence can identify specific molecular changes in the process of tumor occurrence and development and predict the patient’s prognosis by processing a large amount of complex multi-omics data, thereby revealing the heterogeneous characteristics of the tumor microenvironment (27–29). It is anticipated that in the future, artificial intelligence will provide substantial technical support for the study of GBM metabolism and establish the foundation for future precision medicine and personalized treatment strategies.

Bibliometrics, which originated in the early 20th century, is a branch of scientometrics in informatics and refers to the interdisciplinary science of quantitative analysis of all knowledge carriers using mathematical and statistical methods (30). The application of bibliometrics can assist scientists and clinicians in identifying the research lineage and emerging trends within a specific research area (31–33). This enables a deeper understanding of the knowledge structure within the field of research. To date, several bibliometric articles about GBM have been published (34–37), yet there is no bibliometric study focused on GBM metabolism. The objective of this study is to provide a comprehensive analysis and visualization of research related to glioblastoma (GBM) metabolism, encompassing lipid, amino acid, nucleotide, and sugar metabolism, from 2014 to 2024. This analysis aims to evaluate the current research status in the field of GBM metabolism and identify potential future trends and research hotspots.

This study is based on WoSCC data and uses tools such as the Bibliometrics online analysis platform, CiteSpace, VOSviewer and Microsoft Excel to analyze the number of published articles on glioblastoma metabolism, countries, authors, institutions, journals, references, keywords, etc. To sort out the directions and hot spots of glioblastoma metabolism research and predict the direction of further research.

## 2 Materials and methods

### 2.1 Data acquisition

**Figure 1:**
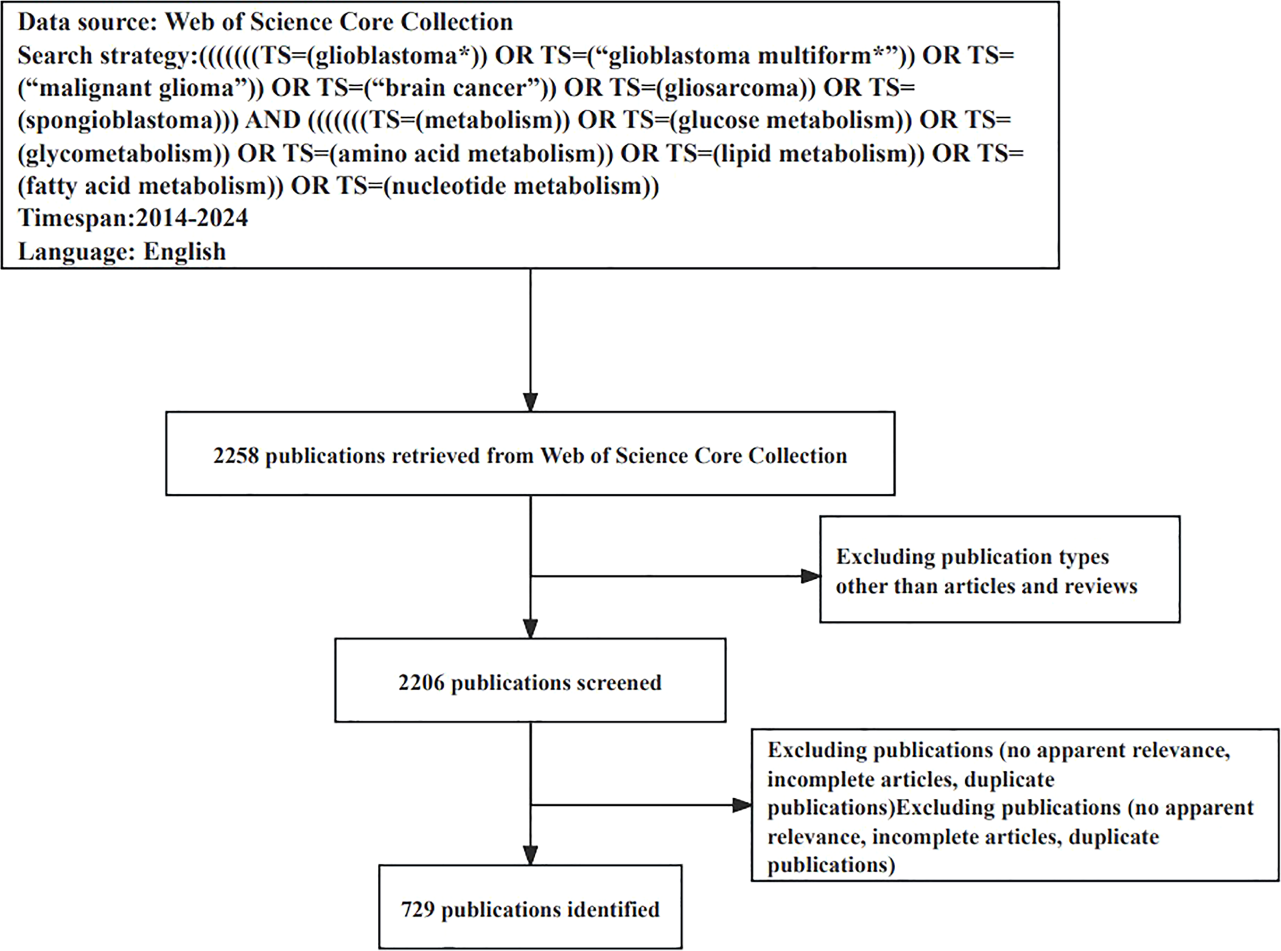
Flowchart of included and excluded publications in the study.

A data search was conducted on 1 May 2024 in the Web of Science Core Collection database (WoSCC) for relevant literature, including articles published between 1 January 2014 and 30 April 2024 related to glioblastoma metabolism. Wildcards were used systematically and scientifically to perform the search, and the specific search strategy is shown in **Table 1**. Only original research articles and review articles published in English-language journals were selected for analysis. Subsequently, two authors undertook an independent assessment of the relevance of each publication, based on an analysis of the title and abstract. Articles that were irrelevant to the study topic, incomplete, retracted, or duplicated were excluded. To mitigate the potential for bias in the analysis, dissenting articles were collectively deliberated upon and a final consensus was reached. The process of literature screening is illustrated in the accompanying **Figure 1**. All information from all the screened literature was saved in plain text file format.

**Table 1:** Search strategies.

Set	Search formula
#1	(((((TS=(glioblastoma*)) OR TS=(“glioblastoma multiform*”)) OR TS=(“malignant glioma”)) OR TS=(“brain cancer”)) OR TS=(gliosarcoma)) OR TS=(spongioblastoma)
#2	((((((TS=(metabolism)) OR TS=(glucose metabolism)) OR TS=(glycometabolism)) OR TS=(amino acid metabolism)) OR TS=(lipid metabolism)) OR TS=(fatty acid metabolism)) OR TS=(nucleotide metabolism)
#3	#1 AND #2

TS, Topic Search.

### 2.2 Bibliometric analysis

The data was processed using Microsoft Office Excel 2016 (Microsoft Corporation, Redmond, WA, USA) to demonstrate the yearly growth trend of publications. CiteSpace (version 6.3.R2) is a citation visualization and analysis software that focuses on and analyses the fundamentals embedded in scientific analysis, developed in the context of scientometrics and data visualization. It is used to perform cluster analysis of keywords, references and other data sets, as well as the generation of timelines to illustrate the most significant citation outbreaks (38, 39). VOSviewer (version 1.6.20) is a computer program for building and viewing bibliometric maps (30, 40). It is employed to conduct co-occurrence analyses of countries/regions, institutions, authors and keywords in a variety of ways. The Bibliometric Analysis Online Platform (https://bibliometric.com/) is an online platform designed for bibliometric analysis. It is intended to assist researchers, academic institutions and policymakers in analyzing and visualizing citation networks and research trends in the academic literature. The platform is employed to illustrate the number of publications in the literature and the number of publications by country.

### 2.3 Ethics in research

The study did not require the consent of the Ethical Medical Council.

## 3 Results

### 3.1 Number and trend analysis of published papers

**Figure 2:**
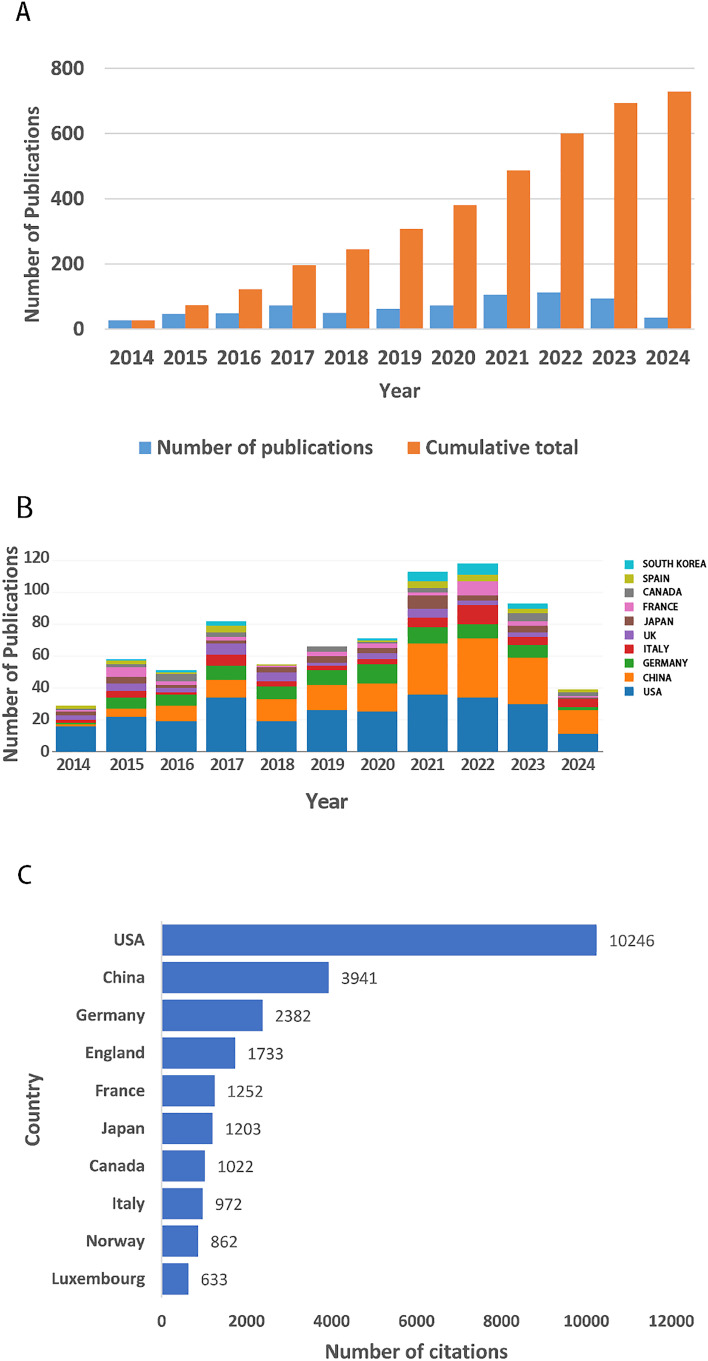
Number and trend of annual publications. **(A)** Annual publication volume and trends, including annual publication and cumulative publication. **(B)** Annual publication trends of the top 10 countries/regions. **(C)** Top 10 countries/regions with the highest total number of citations of publications.

A comprehensive search of the WOScc database revealed a total of 2,258 research publications on GBM metabolism between 1 January 2014 and 30 April 2024. However, data from 2024 could not be fully included due to the search deadline. Following a series of screening processes, a total of 729 documents were ultimately selected for in-depth bibliometric analysis. Of the selected papers, 617 (84.6%) were articles and 112 (15.4%) were reviews. The results of the analyses revealed a clear trend: the number of research articles on GBM metabolism showed an overall increase during the period under review. **Figure 2A** illustrates the trend of publications, both in terms of annual and cumulative figures. Specifically, the number of related studies continued to increase between 2014 and 2017. Although there was a slight decline in 2018, academic interest in research in this field increased again from 2018 until 2022, reaching a peak in 2022, a total of 113 articles published that year. In 2023, 94 high-quality articles were published, although the number of publications declined from the previous year. The data provide ample evidence that GBM metabolism has received widespread academic attention and research, highlighting the importance and development potential of the field.

### 3.2 Distribution of countries/areas and institutions

**Figure 3:**
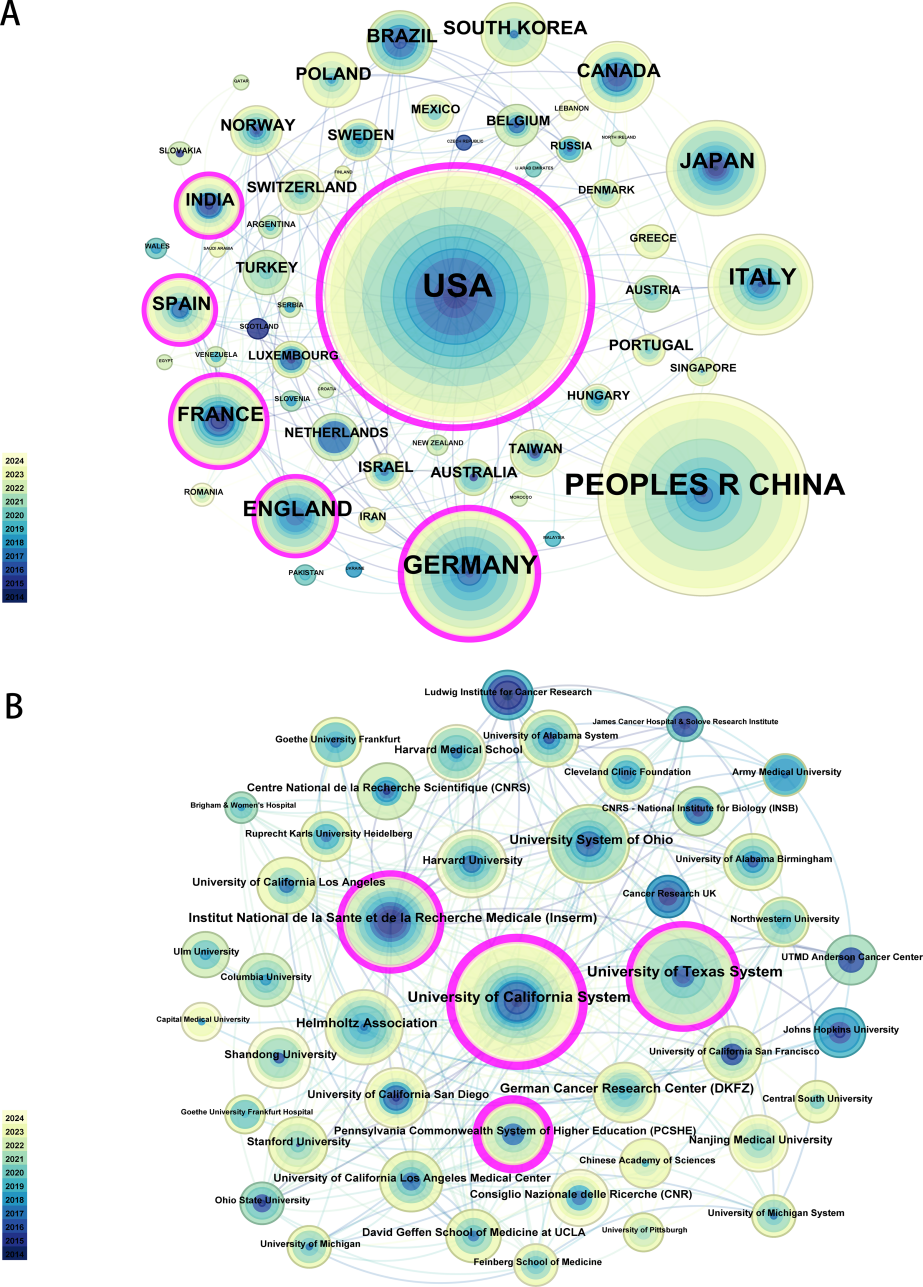
Visualization of collaboration between countries/regions and institutions **(A)** Collaboration between countries. **(B)** Collaboration between institutions. Each circle represents a country/region or institution. The size of the circle represents the publication output of the country/region or institution. The lines between the circles represent the collaboration between countries/regions or institutions. Nodes with high centrality are displayed as purple rings. The thickness of the purple rings represents the size of the betweenness centrality value.

A total of 729 publications were obtained from 245 countries/regions and 3,314 institutions. As shown in **Table 2**, the countries with the highest number of publications were the USA (272, 37.3%) and China (181, 24.8%), followed by Germany (82, 11.2%), Italy (51, 7.0%) and the United Kingdom (38, 5.2%). The countries with the highest centrality in terms of publication are the USA (0.72), followed by Germany (0.33), Spain (0.18) and India (0.15). The United States leads the world in H-Index, with a total of 59, followed by China (37), Germany (31) and Japan (31). As evidenced by the relatively high number of publications and publication centrality, the United States and Germany have established a robust collaborative network with other countries in this field. Conversely, while Italy, the United Kingdom and China also occupy leading positions in terms of the number of publications, their relatively low centrality suggests that these regions engage in relatively limited cooperation with other countries. The H-Index of the United States and China is relatively high, indicating that the quality of articles published by these two countries is widely acknowledged by the academic community. The top 10 institutions in terms of number of published articles are shown in **Table 3**. The research institution with the highest number of publications was the University of California System (53), followed by the University of Texas System (37), Helmholtz Association (31) and German Cancer Research Center (DKFZ) (26). Among the top ten institutions in terms of number of publications, the University of California System (0.25) has the highest centrality, followed by Pennsylvania Commonwealth System of Higher Education (PCSHE) (0.23), Institut National de la Sante et de la Recherche Medicale (Inserm) (0.15) and University of Texas System (0.12). The University of California System has the highest H-Index, with a total of 25, followed by the University of Texas System (21) and the Helmholtz Association (19). **Figure 3** shows a visual mapping of collaborative relationships between countries/regions and institutions. A node represents an institution. The size of the node is indicative of the number of publications and the institution’s centrality.

**Table 2:** Top 10 countries/regions regarding number of publications, the corresponding frequency of citations and centrality.

Rank	Countries/regions	Publications	Percentage	Citations	Centrality	H-Index
1	USA	272	37.3%	10246	0.72	59
2	China	181	24.8%	3941	0.06	37
3	Germany	82	11.2%	2382	0.33	31
4	Italy	51	7.0%	972	0.02	20
5	England	38	5.2%	1733	0.14	20
6	Japan	36	4.9%	1203	0.03	31
7	France	33	4.5%	1252	0.14	17
8	Canada	25	3.4%	1022	0.07	15
9	Spain	24	3.3%	594	0.18	15
10	South Korea	22	3.0%	362	0.00	12

**Table 3:** Top 10 institutions by number of publications.

Rank	Institution	Publications	Centrality	H-Index
1	University of California System	53	0.25	25
2	University of Texas System	37	0.12	21
3	Helmholtz Association	31	0.08	19
4	German Cancer Research Center (DKFZ)	26	0.05	17
5	Institut National de la Sante et de la Recherche Medicale (Inserm)	25	0.15	17
6	University System of Ohio	22	0.07	17
7	Harvard University	19	0.08	17
8	University of California San Diego	17	0.07	14
9	Pennsylvania Commonwealth System of Higher Education (PCSHE)	15	0.23	11
10	University of California Los Angeles	15	0.01	14

### 3.3 Analysis of co-author networks and core author distribution

A total of 5420 authors contributed to 729 selected articles related to GBM metabolism. Among all authors, Mischel PS emerged as the most prolific author with 14 publications (1.92% of all publications), closely followed by Cloughesy TF (11, 1.50%) and Siegelin MD (11, 1.50%), with Chinnaiyan P (10, 1.37%), Karpel- Massler G (10, 1.37%) and Westhoff MA (10, 1.37%) tied for fourth. The top 10 authors with the most publications are shown in **Table 4**. The ten most prolific authors published 102 papers, representing 14.0% of the total number of papers. In addition, the H-Index of these authors is greater than or equal to 7.

**Table 4:** The authors with the greatest number of publications and citations.

Rank	Most of the published authors	Publications	H-Index	Most of the cited authors	Citations	H-Index
1	Mischel PS	14	11	Guo DL	104	7
2	Cloughesy TF	11	9	Chakravarti A	100	6
3	Siegelin MD	11	10	Stoll EA	93	2
4	Chinnaiyan P	10	9	Geng F	91	6
5	Karpel-Massler G	10	10	Nakano I	90	8
6	Westhoff MA	10	10	Agnihotri S	89	6
7	Masui K	9	7	Cheng X	87	5
8	Shu C	9	9	Wu XN	87	5
9	Nguyen TTT	9	8	Mischel PS	85	11
10	Wang J	9	7	Chinnaiyan P	82	9

**Figure 4:**
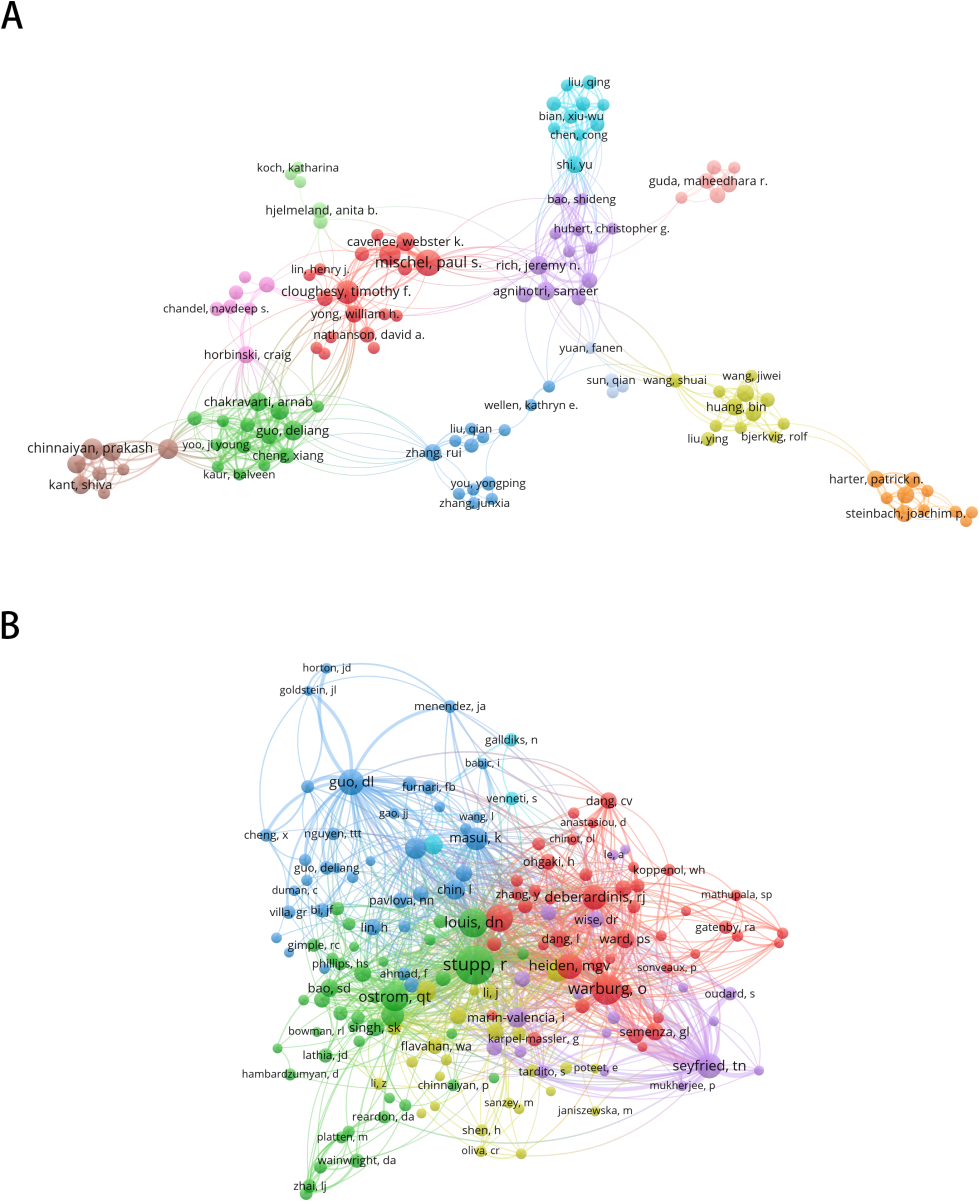
Visual map of the author collaboration network. **(A)** Collaboration graph of 266 authors who have published more than 3 papers. **(B)** Collaboration graph of 168 authors who have been cited more than 20 times. Node and font size are positively correlated with the number of published articles, and the thickness of the connecting line represents the density of collaboration between authors.

In terms of citations, the authors with the highest number of citations are presented in **Table 4**. Guo DL received the highest number of citations (104), followed by Chakravarti A (100 citations), Stoll EA (93 citations), Geng F (91 citations) and Nakano I (90 citations). Except for Stoll EA (The author has only published two articles in this field), the H-Index of these authors is equal to or greater than 5. The successful completion of a research project often depends on the collaboration of several researchers. By analyzing in-depth collaborative networks between authors, we can identify key researchers in a given academic field and assess how closely they work together. **Figure 4** presents a visual representation of the author collaboration network. **Figure 4A** focuses on the 266 researchers who have published more than three papers, while **Figure 4B** depicts the collaboration graph of 168 authors who have been cited more than 20 times. Node and font size are positively correlated with the number of published articles, and the thickness of the connecting lines represents the density of collaboration between authors. Our findings indicate that these prolific authors were primarily from countries or organizations that were highly prolific and had established close collaborative networks between them.

### 3.4 Number of journal publications and journal impact analysis

The value of journal research lies in the identification of research hotspots and disciplinary branches in a particular field, as well as in revealing the layout of key journals in the field, providing indispensable reference materials for academic researchers. The Impact Factor (IF) is a key quantitative metric employed to assess the academic importance of a journal and the quality of its papers. Furthermore, the Impact Factor is employed as a gauge of a journal’s practical applicability and as a means of demonstrating its academic impact. The concept was first proposed in 1972 by Eugene Garfield, the founder of the Institute for Scientific Information (ISI) in the United States, and has since become an important evaluation tool in international academic publishing. In this study, we conducted a meticulous selection and inclusion of 729 articles from 285 different journals. With the assistance of a bibliometric online analysis platform, we were able to provide insights into the impact of these journals. Specifically, **Table 5** demonstrates the top ten journals in terms of the total number of articles published and their impact on academia, while **Table 6** reveals the top ten journals in terms of the total number of citations and their impact on academia. The data presented here provide valuable insights into the academic standing and influence of the journals in question. The International Journal of Molecular Sciences was the journal with the highest number of articles published, with a total of 37 articles. This was followed by Cancers (34) and Oncotarget (31). In terms of the number of citations, Neuro-Oncology emerged as the most cited journal, with a total of 218 citations. This was followed by Oncotarget (85 citations) and Clinical Cancer Research (81 citations). Furthermore, the Cell journal had the highest average number of citations per article (51), followed by Nature Cell Biology (44), Embo Journal (21) and Nature Neuroscience (21). The H-Index of these journals is greater than or equal to 9. In our study, we identified several journals that have a high impact on the academic world. These journals demonstrated a high level of productivity, with a significant number of articles published and citations received. These results provide important insights into the status and influence of journals in academia.

**Table 5:** Top 10 journals by number of articles published.

Rank	Journal	Publications	JCR	IF	H-Index
1	International Journal of Molecular Sciences	37	Q2	4.9	14
2	Cancers	34	Q2	4.5	11
3	Oncotarget	31	NA	NA	23
4	Neuro-Oncology	25	Q1	16.4	17
5	Frontiers in Oncology	18	Q3	3.5	11
6	Scientific Reports	18	Q2	3.8	11
7	Cells	17	Q2	5.1	9
8	Cell Death & Disease	16	Q1	8.1	11
9	Plos One	13	Q3	2.9	11
10	Clinical Cancer Research	11	Q1	10.0	10

N/A = Oncotarget is no longer included in the JCR partition table and so is not applicable.

**Table 6:** Top 10 journals by number of citations.

Rank	Journal	Citations	JCR	IF	H-Index
1	Neuro-Oncology	218	Q1	16.4	17
2	Oncotarget	85	NA	NA	23
3	Clinical Cancer Research	81	Q1	10.0	10
4	Cancers	64	Q2	4.5	11
5	Cell Death & Disease	60	Q1	8.1	11
6	Frontiers in Oncology	57	Q3	3.5	11
7	Plos One	55	Q3	2.9	11
8	Frontiers in Cell and Developmental Biology	52	Q2	4.6	3
9	Journal of Clinical Investigation	51	Q1	13.3	4
10	Cell	51	Q1	45.5	1

N/A = Oncotarget is no longer included in the JCR partition table and so is not applicable.

### 3.5 Co-citation analysis of reference

A total of 31,358 references were cited in the 729 articles selected for this search. The 10 most frequently cited references have been listed in detail in **Table 7**. These highly cited articles were published in high-quality journals: New England Journal of Medicine (IF=96.2), Nature (IF=50.5), Cell (IF=45.5), Science (IF=44.7) and Lancet Oncology (IF=41.6) and Cancer Cell (IF=48.8). The 2005 publication by Roger Stupp’s team of “Radiotherapy plus concomitant and adjuvant temozolomide for glioblastoma” is the document most frequently cited in the field, with a total of 2,324 citations.

**Table 7:** The top 10 most frequently cited references.

Rank	Title	Citations	Journal	JCR	IF	DOI
1	Radiotherapy plus concomitant and adjuvant temozolomide for glioblastoma	160	New England journal of medicine	Q1	96.2	10.1056/nejmoa043330
2	Hallmarks of cancer: the next generation	128	Cell	Q1	45.5	10.1016/j.cell.2011.02.013
3	Understanding the Warburg effect: the metabolic requirements of cell proliferation	120	Science	Q1	44.7	10.1126/science.1160809
4	Integrated genomic analysis identifies clinically relevant subtypes of glioblastoma characterized by abnormalities in PDGFRA, IDH1, EGFR, and NF1	102	Cancer Cell	Q1	48.8	10.1016/j.ccr.2009.12.020
5	The somatic genomic landscape of glioblastoma	89	Cell	Q1	45.5	10.1016/j.cell.2013.09.034
6	On the origin of cancer cells	84	Science	Q1	44.7	10.1126/science.123.3191.309
7	Comprehensive genomic characterization defines human glioblastoma genes and core pathways	74	Nature	Q1	50.5	10.1038/nature07385
8	The 2016 World Health Organization Classification of Tumors of the Central Nervous System: A Summary	72	Acta Neurochirurgica	Q3	1.9	10.1007/s00401-016-1545-1
9	Effects of radiotherapy with concomitant and adjuvant temozolomide versus radiotherapy alone on survival in glioblastoma in a randomized phase III study: 5-year analysis of the EORTC-NCIC trial	71	Lancet Oncology	Q1	41.6	10.1016/s1470-2045(09)70025-7
10	Analysis of tumor metabolism reveals mitochondrial glucose oxidation in genetically diverse human glioblastomas in the mouse brain *in vivo*	69	Cell Metabolism	Q1	27.7	10.1016/j.cmet.2012.05.001

**Figure 5:**
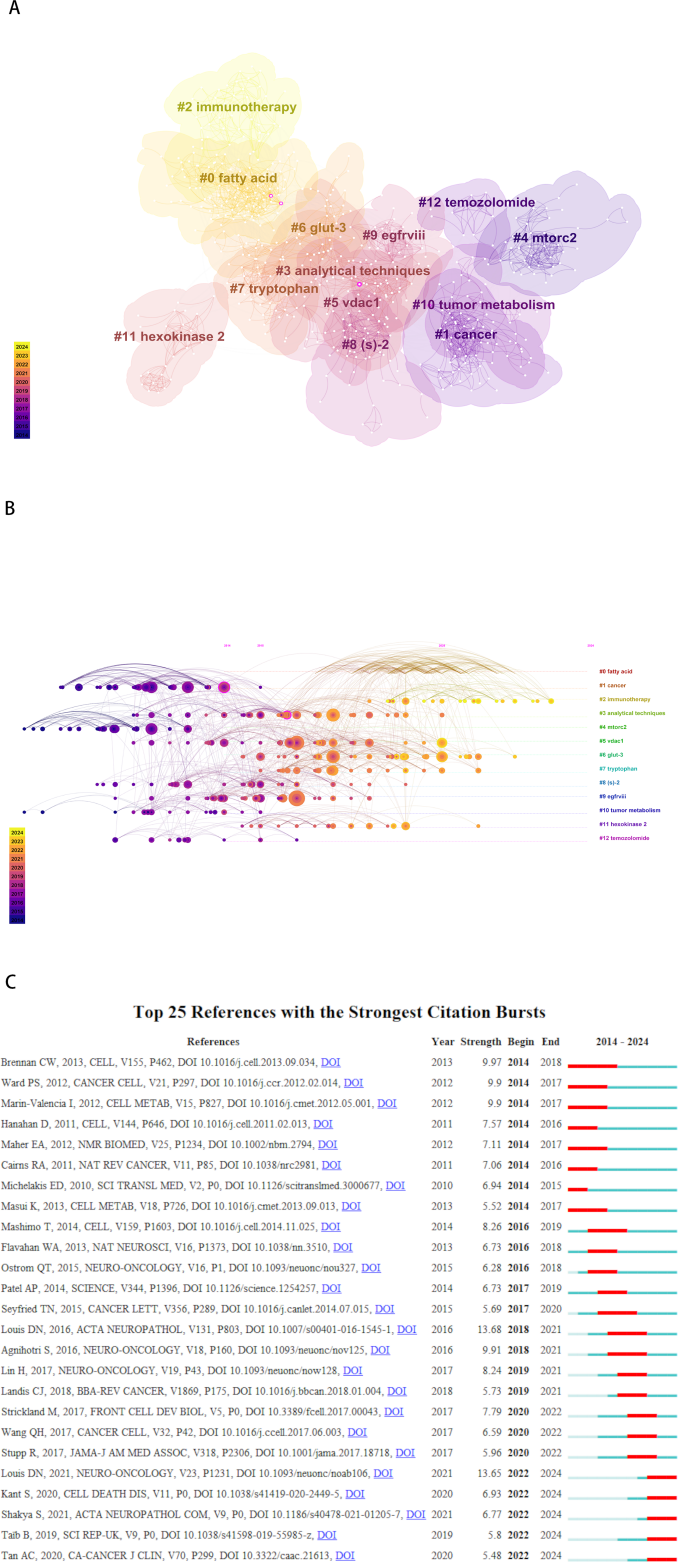
A cluster network analysis of GBM metabolism research publications between 2014 and 2024. **(A)** A visual analysis of the co-citation literature clustering network. The top 13 largest citation clusters are presented. The greater the yellow, the more frequently the references in these clusters have been cited in recent years. **(B)** Timeline view of co-citation references. Each horizontal line represents a cluster, and the smaller the label number is, the larger the cluster the node size reflects the co-citation frequency, while the links represent the co-citation relationship. The colors of the nodes and lines represent the different years of citation. **(C)** The top 25 most cited references.

A network analysis of publication clusters for GBM metabolism-related research between 2014 and 2024 is presented in **Figure 5**. **Figure 5A** illustrates the CiteSpace visualization analysis of the clustered network of co-cited literature. The top 13 largest citation clusters are presented. The number of cluster labels is inversely proportional to the number of articles in each cluster. The greater the number of yellow clusters, the more frequently the references within them have been cited in recent years. The fields of immunotherapy and fatty acids have become a key focus in recent years. **Figure 5B** presents a timeline view of the co-cited references related to GBM metabolism. Each circle represents a major cited reference in the cluster, while the size of the circle on the timeline indicates the frequency of citations. Links indicate the co-citation relationship and the colors of the nodes and lines represent the different citation years. Cancer, tumor metabolism and morc2 appeared earlier in the field, while immunotherapy has been a hot research topic in recent years.

A burst of citation frequency is defined as a sudden and significant increase in the number of citations a document receives over a period, which is higher than the average for that document. Such analysis can assist in comprehending the alterations in research focus over time and investigating the prevailing trends and research interests within the research domain. **Figure 5C** illustrates the 25 most cited references over the period between 2014 and 2024. The red bars represent high citation frequencies, while the blue bars represent lower citation frequencies. Among the most cited articles, the reference with the strongest citation burst intensity (intensity = 13.68, burst period = 2018-2021) is the article published by Louis DN et al. in 2016 in Acta Neuropathologica under the title ‘The 2016 World Health Organization Classification of Tumors of the Central Nervous System: a summary’, which ranked 8th among the most cited articles.

### 3.6 Keyword visual analysis

**Figure 6:**
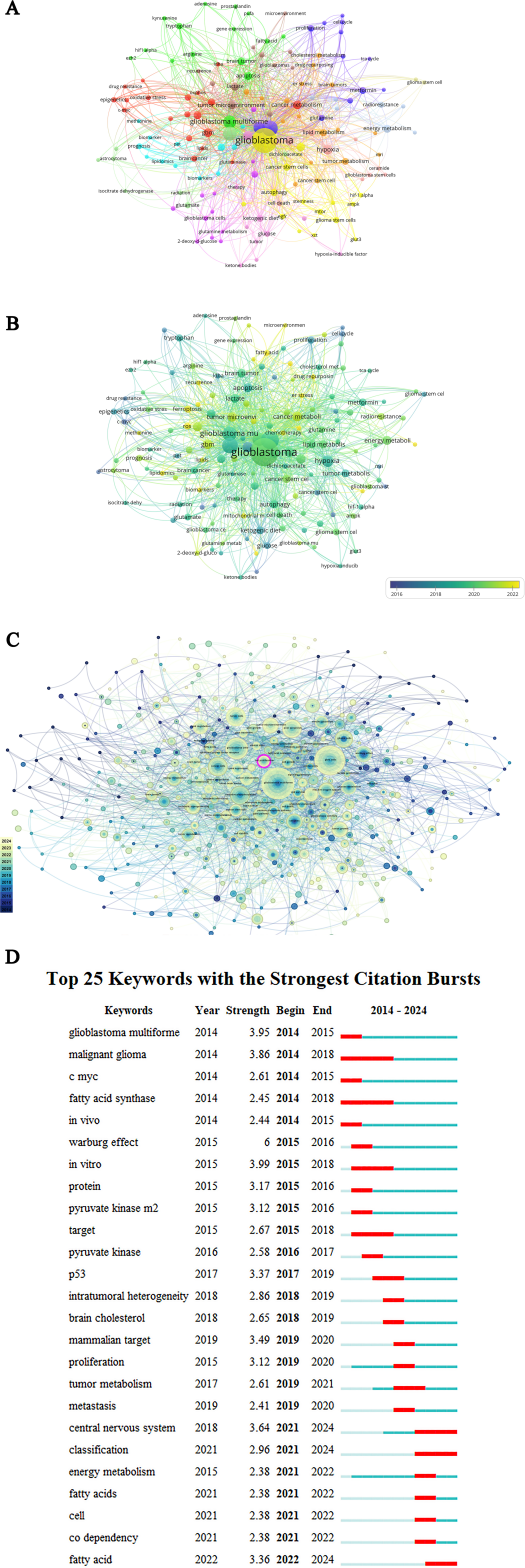
A network visualization of keyword/hot topics co-occurrence. **(A)** Network graph of keyword co-occurrence. **(B)** Time view of keywords. **(C)** Visualization of popular research topics. **(D)** The top 25 keywords with the highest citation burst rate.

The co-occurring keyword network and overlay visualization are presented in **Figure 6**. The ten most frequent keywords were as follows: “glioblastoma”, “metabolism”, “glioma”, “glycolysis”, “glioblastoma multiforme”, “hypoxia”, “Warburg effect”, “tumor microenvironment”, “cancer” and “cancer metabolism”. **Figure 6A** shows the keywords divided into 13 clusters. The largest cluster, which is red, is related to the metabolic reprogramming of brain tumor cells, including “brain cancer”, “lipids”, “mass spectrometry metabolic reprogram metabolomic” and “oxidative stress”. The second major cluster is orange and is related to lipid metabolism in brain tumors, including “brain tumors”, “cholesterol metabolism”, “lipid metabolism” and “fatty acids”. The third cluster is yellow and is related to mitochondrial metabolism in glioblastoma stem cells. It includes the keywords “cancer stem cells”, “glioblastoma”, “mitochondrial metabolism” and “autophagy”.

**Figure 6B** also shows the evolution of the GBM metabolism research clusters over time. The size of the circle and the font represent the frequency of the word, and the lines indicate the correlation between them; the purple color indicates that the keyword appeared on average in 2016 or even earlier, while the yellow color indicates that the keyword appeared in the last three years after 2022. In GBM metabolism research (2014-2024), the research trend has shifted from the ketogenic diet and Warburg effect to fatty acid metabolism and iron metabolism. In addition, energy metabolism has been a hot direction of much attention in this field.

Keyword Burst refers to the frequency of occurrence of a keyword over a specified period, indicating an elevated level of interest or activity. By calculating the strength of a keyword burst, it is possible to gain insight into the current hotspots and future development trends within a given research field. Researchers can then use this information to inform their research direction and identify valuable sources of information. **Figure 6D** illustrates the top 25 keywords with the strongest citation bursts. The keywords marked with red bars indicate a sudden surge in the frequency of use of the keyword within that period, while the blue color indicates a relatively less cited time. The “Warburg effect” has the highest outburst intensity (6.00), followed by “*in vitro*” (3.99), “glioblastoma multiforme” (3.95), “malignant glioma” (3.86) and “central nervous system” (3.64). The central nervous system, classification and fatty acid show strong citation bursts in 2024. In conjunction with **Figure 6C**, glioblastoma, cell proliferation and fatty acids remain prominent in investigation within GBM metabolism.

### 3.7 Research discipline analysis

**Figure 7:**
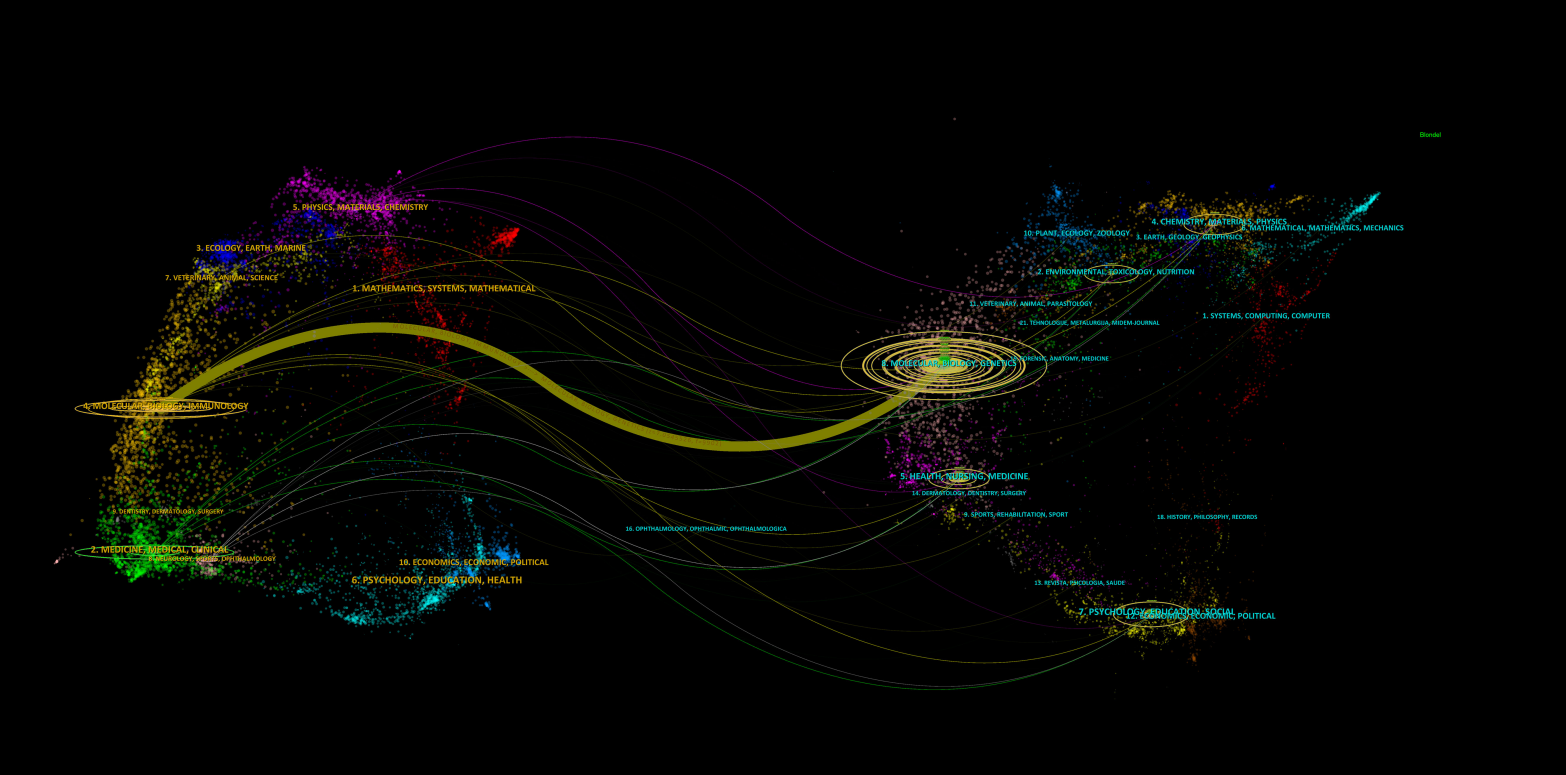
A dual-map overlay of journals on GBM metabolism.

The dual map overlay of journals can be utilized to illustrate the distribution of subjects across academic journals, the evolution of citation trajectories and the relocation of research centers (41). Research on GBM metabolism encompasses a multitude of disciplines and fields. **Figure 7** presents a dual-map overlay of journals on GBM metabolism. The clusters on the left represent the collection of citing journals, which serve to illustrate the current state of knowledge; the clusters on the right represent the collection of cited journals, which demonstrate the knowledge base that is currently being cited by the frontier of knowledge. The length of the ellipse is indicative of the number of authors, while the width is indicative of the number of publications. The curve connecting the two sides represents the connection between citations. The use of different colors represents the distinction between subject areas. The greater the thickness of the curve, the stronger the connection. As illustrated in the figure, the citing journals are predominantly distributed across subjects #2 (Medicine, Medical, Clinical) and #4 (Molecular, Biology, Immunology), while the cited journals are primarily concentrated in subject #8 (Molecular, Biology, Genetics). Additionally, a subset of the cited journals are also distributed across subjects #2 (Environmental, Toxicology, Nutrition), #4 (Chemistry, Materials, Physics), #5 (Health, Nursing, Medicine) and #7 (Psychology, Education, Social). The literature in the disciplines of Molecular, Biology and Immunology is significantly affected by the disciplines of Molecular, Biology and Genetics (z = 7.05, f = 9902).

## 4 Discussion

Data from 729 articles on GBM metabolism screened in Web of Science from 2014 to 2024 were analyzed using CiteSpace 6.3.R2 Advanced, VOSviewer 1.6.20, and Microsoft Office Excel 2016. Based on data, we assessed country/institution distribution, author contributions, core articles and research hotspots.

### 4.1 General distribution

The analysis of this study is based on 729 articles on glioblastoma metabolism from 245 countries, 3,314 institutions, and 5,420 authors, published between 1 January 2014 and 30 April 2024 in the Web of Science Core Collection (WoSCC) database. The rapid increase in the number of articles indicates that GBM metabolism is receiving more and more attention. In 1920, the German biologist Otto Warburg proposed the ‘Warburg effect’, which states that under normal, aerobic conditions, most healthy cells completely oxidize glucose in the mitochondria through the tricarboxylic acid cycle and the electron transport chain, thereby obtaining the maximum amount of energy (ATP). In contrast, cancer cells rely primarily on glycolysis for energy production even in the presence of sufficient oxygen (42–44). Since that time, the scientific community has initiated studies related to the metabolism of tumor cells. The number of studies related to GBM metabolism has increased steadily over the past decade. The year with the highest number of publications, 2022, will have approximately four times as many publications as 2014.

In terms of the most productive countries, the United States was the most prolific, followed by China, Germany, Italy, the United Kingdom, Japan and France, all of which published more than 30 articles (**Figure 2**). The top three countries with the highest number of published articles were the United States, China and Germany, which collectively published 535 articles, accounting for 73.39 percent of all articles. Furthermore, the top three countries with the highest frequency of collaboration were the United States, Germany and Spain. The countries with the highest number of article citations are the United States, China, Germany, the United Kingdom, France, Japan and Canada, all of which have been cited more than 1,000 times (**Figure 2**). The above findings demonstrate the significant contribution and leading position of the United States, China and Germany in the research of GBM metabolism. This may be attributed to their adequate research funding, advanced research infrastructure, and well-established academic publishing and evaluation system, which attracts outstanding scholars from all over the world to pursue further studies in the region. In terms of the most productive research institutions, the University of California System is at the pinnacle of the list, followed by the University of Texas System, the Helmholtz Association, the German Cancer Research Center (DKFZ), Institut National de la Sante et de la Recherche Medicale (Inserm) and the University System of Ohio, all of which have published more than 20 publications (**Figure 2**).

In terms of the most influential journals and authors, the International Journal of Molecular Sciences (37 articles) and Cancers, Oncotarget and Neuro-Oncology (all published more than 20 articles) are the most prominent (**Table 5**). The Oncology journal published 25 articles that received the most citations (218 citations), followed by Oncotarget (85 citations) and Clinical Cancer Research (81 citations). The Journal Citation Reports (JCR) is one of the most common metrics used to assess the impact of journals. It divides all journals into four quartiles (Q1-Q4) based on their impact factor. Of the top ten most cited journals, Q1 journals account for 50 percent (**Table 6**). As illustrated in **Table 7**, the majority of the most highly cited articles were published in journals with high impact factors. The high impact factor of a journal is often indicative of the quality of the research published therein, which has typically undergone rigorous peer review. This is a crucial factor in the results of our bibliometric analysis. Journals with high impact factors typically exert greater academic influence and can attract a larger readership and a greater number of citations, thereby exerting a more significant impact on the field of GBM metabolism-related research. To illustrate, the New England Journal of Medicine has an impact factor of 96.2, which evinces its preeminent status within the domain of biomedicine. Consequently, research findings about GBM metabolism published in this journal receive heightened attention and citations.

Mischel PS is one of the most prolific authors in the field, with 14 publications and 85 citations, which attests to his significant impact in the field of glioblastoma metabolism. Other distinguished authors include Cloughesy TF and Siegelin MD On the other hand, Guo DL ranks first in terms of citation rankings, having been cited 104 times. Other authors who have been cited more than 90 times include Chakravarti A., Stoll EA, Geng F and Nakano I. These authors and their teams have made significant contributions to the field of GBM metabolism. The results of their valuable research have provided us with important references for our future research in this field.

In 2005, physicist Jorge E. Hirsch proposed the concept of the H-index (45), which is a concise and effective indicator for measuring the quality and influence of the research results of scientists, academic institutions, or countries. It is based on the number of papers published by researchers and the number of citations of these papers, to comprehensively reflect the productivity and academic influence of researchers. At the national level, the United States, China, Germany and Japan all have relatively high H-Indexes, indicating that the scientific research results of these countries are not only substantial in number, but also exert a considerable academic influence on the scientific research field of GBM metabolism. In particular, they have exerted a powerful, autonomous, and unavoidable influence in high-quality, widely cited fields. At the institutional level, the H-Index of the University of California System, the University of Texas System and the University of Texas System are among the highest. Despite the relatively limited number of publications, Harvard University’s H-Index remains notably high, suggesting that the impact of its research output is concentrated in a small number of high-quality papers. The research conducted by Harvard University is often more refined, and its high-level scientific research results frequently exert an inevitable impact on core areas. At the author level, Mischel PS is one of the authors with the highest number of published papers and is also ranked highly among the most highly cited authors. The high H-Index reflects his sustained contributions to the field of GBM metabolism and the inevitable impact of these contributions within the literature network. While Guo DL’s number of published papers is not among the top ten, his relatively high H-Index suggests that the quality and citation rate of his papers in the field of GBM metabolism are comparatively high.

Concerning subject areas, the dual-map overlay of journals demonstrates the current state of knowledge flow and interdisciplinary collaboration. The most recent research findings on GBM metabolism are primarily disseminated in journals belonging to the following disciplines: medicine, clinical medicine, molecular science, biology, immunology and other related fields. Conversely, research findings published in molecular science, biology and genetics, as well as health, nursing, medicine, psychology, education and sociology provide a robust foundation for contemporary research endeavors. Research on GBM metabolism is inherently multidisciplinary and cross-disciplinary. The close communication and mutual influence between different disciplines not only introduce new ideas and new methods into this field but also provide impetus for in-depth research and breakthroughs. The advancement of fundamental science (such as molecular science, biology, genetics, etc.) furnishes theoretical substantiation for comprehending the pertinent mechanisms of GBM metabolism. Meanwhile, applied disciplines, such as medicine and clinical medicine, to a certain extent, are capable of transforming these fundamental research outcomes into clinical practice. It is this interdisciplinary collaboration and therapeutic integration that has driven the continuous advancement of GBM metabolic research and provided solutions for future targeted and precise treatments.

### 4.2 Hotspots and Frontiers

The selection of keywords is a crucial aspect of literature management and scholarly communication. These words are carefully chosen for their ease of retrieval and comprehension of the core content of a paper, and can succinctly reflect the core theme and discussion points of the paper. Through in-depth analysis of keywords, it is possible to summarize the research focus of a particular subject area, identify current research hotspots and possible future development trends, and assist academics in tracking the dynamics of the discipline and advancing knowledge. In our study, the keywords that appear more frequently are “glioblastoma”, “metabolism”, “glycolysis”, “hypoxia”, “Warburg effect”, “cancer metabolism”, “mitochondria”, “tumor microenvironment”, “energy metabolism” and “metabolic reprogramming”. These keywords are mainly related to GBM cellular glucose metabolism, suggesting that they are some of the more popular topics in the last decade. In recent years, several new keywords have emerged in the field of cancer research, including “Fatty acids”, “mitochondrial metabolism”, “iron metabolism”, “tumor microenvironment”, “metabolism”, “tumor heterogeneity”, “cancer metabolism”, “immunotherapy”, “arginine” and “cholesterol”, which are mainly focused on the metabolism of lipids and the heterogeneity of GBM cells. This suggests that the above keywords have become a new and popular topic in recent years.

As is the case with other types of tumor cells, GBM cells also demonstrate a significant Warburg effect. Even in an aerobic environment, they continue to obtain energy through glycolysis rather than through the more efficient oxidative phosphorylation pathway (13). This metabolic mode allows tumor cells to obtain the energy and metabolites necessary for growth rapidly. This significant difference in sugar metabolism between GBM cells and normal cells has initiated a new research direction for researchers. At present, a considerable number of scientific research teams have initiated research in this area, and have achieved notable scientific research results. For instance, the research team led by Zhenxing Zhang discovered that the protein DHHC9 stimulates glycolysis in GBM cells, thereby promoting their proliferation and tumorigenesis, which is strongly correlated with the patient’s prognosis, indicating that it has significant potential as a therapeutic target and prognostic indicator for abnormal glucose metabolism in GBM cells (21). Rui Yang and his team demonstrated that the transcription factor homeobox A3 (HOXA3) promotes aerobic glycolysis in GBM cells by activating the transcription of lysine-specific demethylase KDM6A, thereby promoting tumor growth (22).

In recent years, abnormalities in lipid and iron metabolism have emerged as a significant area of concern for researchers in the field of GBM research, with the potential to become new therapeutic targets. Compared with normal cells, GBM cells exhibit aberrant lipid metabolism, which enables them to meet the energy demands of tumor cells (14, 46, 47). Ferroptosis is a form of programmed cell death that arises from the accumulation of iron-dependent lipid peroxides within cells, which is involved in the malignant progression of glioblastoma (48). When intracellular iron levels increase, polyunsaturated fatty acids on the cell membrane are oxidized to produce lipid peroxides, which in turn leads to cell membrane rupture and cell death (49, 50). Jenna K Minami’s team found that knocking down CDKN2A can promote lipid peroxidation in GBM cells, selectively induce tumor ferroptosis, and thereby inhibit the malignant progression of glioblastoma (13). Yang Jiang and colleagues demonstrated that circLRFN5 can facilitate ferroptosis by binding to PRRX2 protein and inducing its degradation, thereby inhibiting the growth of glioblastoma (48). Xiang Cheng and colleagues demonstrated that elevated diacylglycerol acyltransferase 1 (DGAT1) facilitates the storage of surplus fatty acids as triglycerides and lipid droplets, thereby safeguarding GBM from oxidative stress and cell death. Consequently, it can be postulated that the inhibition of DGAT1 represents an efficacious strategy for the limitation of GBM development (11).

The field of glioblastoma metabolism is attracting increasing attention from scholars, which indicates that this field has considerable potential for the diagnosis and treatment of GBM. To achieve this, it is necessary to conduct further research into the mechanisms underlying the abnormal metabolism of glioblastoma cells, and it is expected to provide important references for the diagnosis and treatment of other malignant tumors.

### 4.3 Challenges and prospects

The field of GBM metabolic reprogramming has emerged as a prominent area of interest and a focal point for numerous researchers, suggesting that it holds significant promise in the diagnosis and treatment of GBM. Nevertheless, the process of studying tumor cell metabolic reprogramming presents several significant challenges. Firstly, as cancer progresses, the metabolic phenotype of cancer cells undergoes a corresponding change (51), leading to drug resistance during targeted drug therapy and consequently affecting the patient’s prognosis. Secondly, Secondly, The development of targeted drugs related to metabolism necessitates a robust foundation in medicinal chemistry, structural biology, pharmacokinetics and pharmacodynamics. This represents a significant challenge for researchers, necessitating close collaboration between researchers from diverse disciplines. Furthermore, species-specific differences may result in disparate outcomes when developing targeted drugs, from the animal testing stage to the clinical trial stage. This may, in turn, complicate the evaluation of the side effects of targeted drugs that are designed to target the metabolism of human GBM cells (52). Ultimately, the technical intricacy and high expense associated with metabolomics have largely constrained its extensive implementation in clinical research. Nevertheless, metabolic reprogramming is of vital importance in the field of cancer research. Its influence extends beyond the mere occurrence and development of tumors, offering new avenues for therapeutic intervention. In the future, methods for evaluating and quantifying the metabolic phenotype of human GBM will continue to evolve, including metabolomics, isotope tracing studies and metabolic imaging (28, 53, 54). Concurrently, the integration of multi-omics data and AI-assisted analysis technology, metabolic dynamic labeling and real-time metabolic monitoring technology, and the combination of metabolism and immunotherapy (27, 52, 55, 56) will facilitate a more profound comprehension of the mechanism of GBM metabolic reprogramming. This, in turn, will contribute to the development of more precise and efficacious treatment strategies, the incorporation of precision medicine into the comprehensive treatment regimen of GBM patients, and the provision of more targeted treatment options to GBM patients, while simultaneously reducing adverse effects and enhancing efficacy.

### 4.4 Limitations

It should be noted that our study is not without limitations. Firstly, the data used in this study were sourced exclusively from the WoSCC database. Despite the extensive and comprehensive nature of the WoSCC database, which is the most commonly used source of publications in bibliometric analyses, there may be relevant literature that is not included in it. Secondly, the articles included in the study were those included in WoSCC from 1 January 2014 to 31 April 2024. This may have resulted in the overlooking of important research that was published before or after this period. It should be noted that the database is continuously updated. Only articles published in English were selected, which may have resulted in the omission of research results in non-English languages. Furthermore, the bibliometric analyses may have been influenced by temporal bias. It is typical for papers published earlier to accumulate more citations. A variety of other factors, such as self-citations, may also have an impact on citation rates. Finally, the article screening process, in which the researchers manually screened for literature that was not relevant to the topic, is open to subjective interpretation and may lead to selection bias.

## 5 Conclusion

This article presents a comprehensive examination of the current status and development trends in the field of glioblastoma metabolism research through bibliometric analysis methods. The findings indicated that the metabolic mechanism of GBM plays a pivotal role in tumor occurrence and development, and has emerged as a significant research focus in recent years. Our analysis identified key areas and cutting-edge research directions in metabolic research, including tumor metabolic reprogramming, the tumor microenvironment, lipid metabolism, and the role of metabolic pathways in treatment resistance. As technology continues to evolve and our understanding of tumor metabolism deepens, it is anticipated that further breakthroughs will be made in the field of GBM metabolism research. It provides a valuable reference point for future GBM metabolic research and establishes the foundation for the development of new metabolic-targeted therapeutic strategies. By deepening our comprehension of GBM metabolism, we aspire to identify more efficacious treatments. Continued, comprehensive investigation of this field will foster new prospects for enhancing patient prognoses and treatment outcomes.

## Data Availability

The original contributions presented in the study are included in the article/supplementary material. Further inquiries can be directed to the corresponding author.
